# 845. Impact of the COVID-19 Pandemic on Routine HIV Screening in an Emergency Department

**DOI:** 10.1093/ofid/ofab466.1040

**Published:** 2021-12-04

**Authors:** Elizabeth A Aguilera, Gilhen Rodriguez, Gabriela P Del Bianco, Gloria Heresi, James Murphy, Samuel Prater

**Affiliations:** The University of Texas Health Science Center at Houston, Houston, TX

## Abstract

**Background:**

The Emergency Department (ED) at Memorial Hermann Hospital (MHH) - Texas Medical Center (TMC), Houston, Texas has a long established screening program targeted at detection of HIV infections. The impact of the COVID-19 pandemic on this screening program is unknown.

**Methods:**

The Routine HIV screening program includes opt-out testing of all adults 18 years and older with Glasgow score > 9. HIV 4th generation Ag/Ab screening, with reflex to Gennius confirmatory tests are used. Pre-pandemic (March 2019 to February 2020) to Pandemic period (March 2020 to February 2021) intervals were compared.

**Results:**

72,929 patients visited MHH_ED during the pre-pandemic period and 57,128 in the pandemic period, a 22% decline. The number of patients tested for HIV pre-pandemic was 9433 and 6718 pandemic, a 29% decline. When the pandemic year was parsed into first and last 6 months interval and compared to similar intervals in the year pre pandemic, 39% followed by 16% declines in HIV testing were found. In total, 354 patients were HIV positives, 209, (59%) in the pre-pandemic and 145 (41%) in the pandemic period.The reduction in new HIV infections found was directly proportional to the decline in patients visiting the MHH-ED where the percent of patients HIV positive was constant across intervals (2.21% vs 2.26%). Demographic and outcome characteristics were constant across the compared intervals.

**Conclusion:**

The COVID -19 pandemic reduced detection of new HIV infections by screening in direct proportion to the reduction in MHH-ED patient visits. The impact of COVID-19 pandemic decreased with duration of the pandemic.

**Disclosures:**

**All Authors**: No reported disclosures

